# Supplementary figures and images for: Thioredoxin Reductase-1 as a Potential Biomarker in Fibroblast-Associated HCT116 Cancer Cell Progression and Dissemination in a Zebrafish Model
Source: Cancers (Basel). 2022 Dec 22;15(1):56. doi: 10.3390/cancers15010056 (PMC9817953; doi:10.3390/cancers15010056)

E-cad

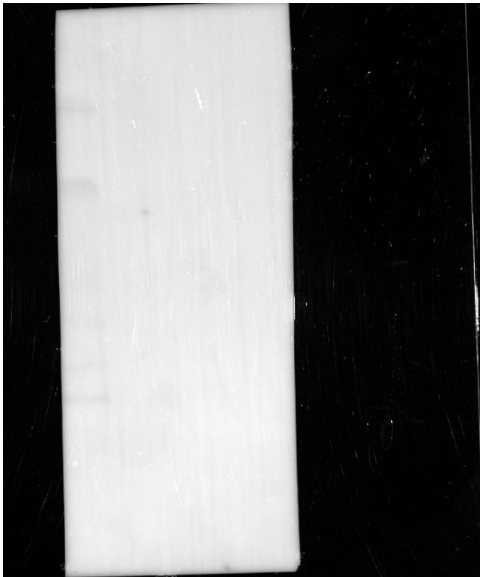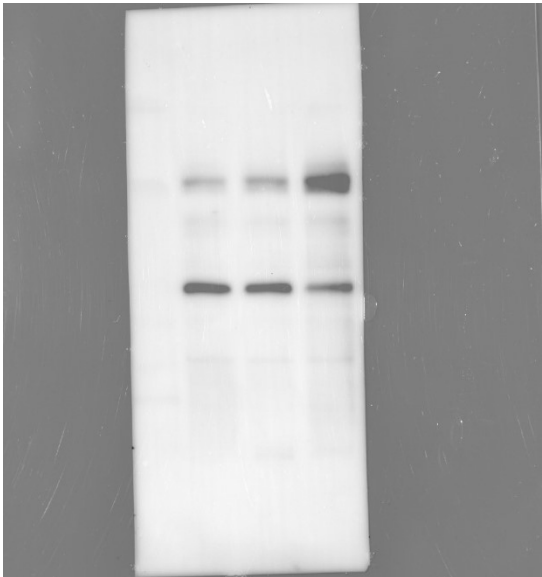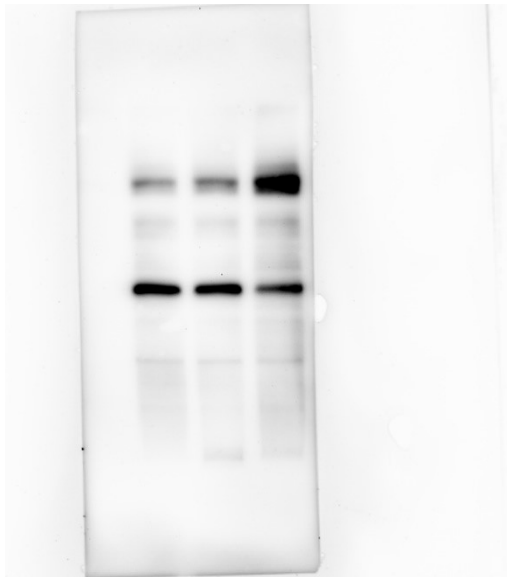

B-actin

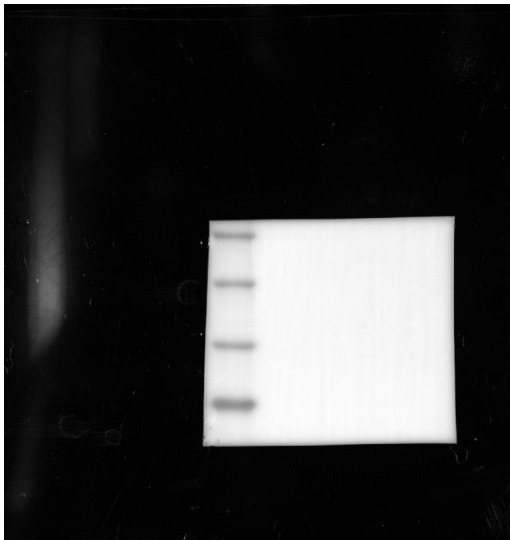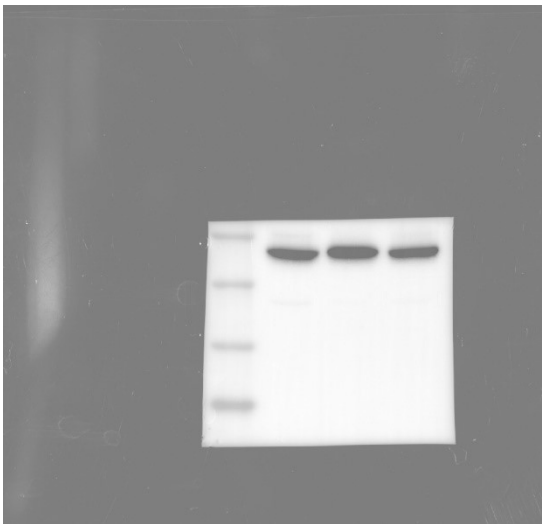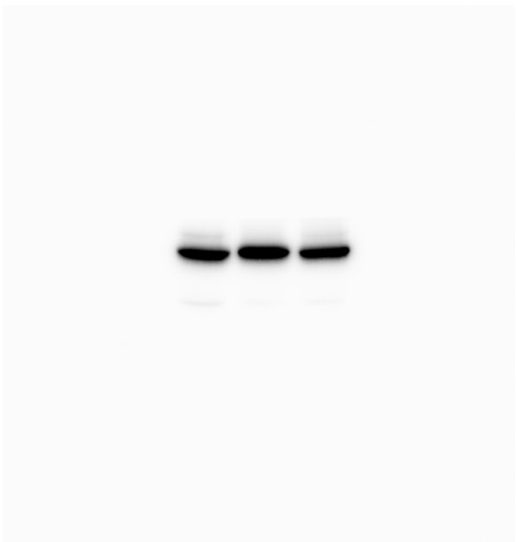

trxr1

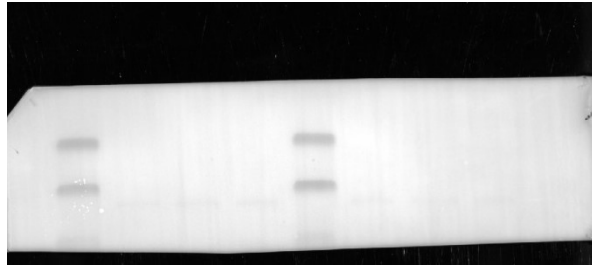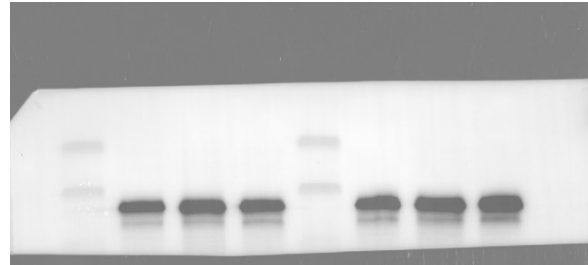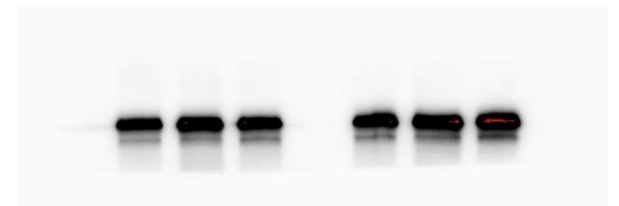

B-actin

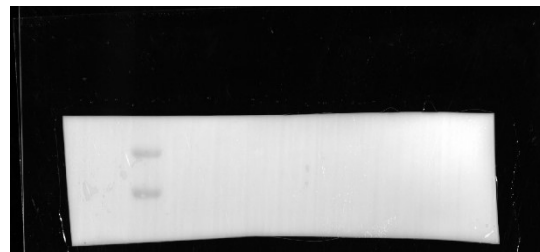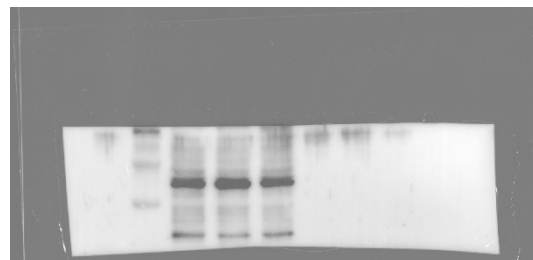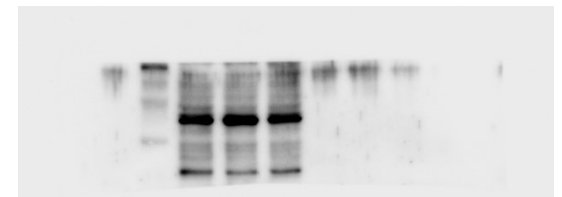

Supplement: Supplementary file 1 [file cancers-15-00056-s001.zip › cancers-2006640-supplementary.pdf]
